# Short-term impact of the Covid-19 pandemic on the global and Turkish economy

**DOI:** 10.3906/sag-2106-271

**Published:** 2021-12-17

**Authors:** Ömer AÇIKGÖZ, Aslı GÜNAY

**Affiliations:** 1 Department of Economics, Faculty of Political Science, Social Sciences University of Ankara, Ankara Turkey

**Keywords:** Covid-19, economy, pandemic, Turkey, world

## Abstract

**Background/aim:**

The Covid-19 pandemic is one of those rare events that affects everyone on earth and changes our lives. The pandemic, which has killed over four million people worldwide, is putting unprecedented pressure on governments to maintain essential health and social services, as well as keep their economies running, even as the virus threatens people’s daily life on every level. Thus, the purpose of this study is to discuss the short-term economic impact of the pandemic by assessing its costs using official economic data for both the world and Turkey. Furthermore, this research highlights possible economic, social, and political pathways for a postpandemic new world.

**Materials and methods:**

This study is a review article that overviews and tracks the economic development of the Covid-19 pandemic from the start, synthesizes and compares current data of reliable institutions, and provides an overall assessment.

**Results:**

The pandemic has certainly caused short-term and long-term damage to economies and living standards for many people. Although there are estimates on what this damage is, the exact degree of the damage is still unknown. However, it seems that the recovery will be gradual, long-lasting, and unpredictable due to the unprecedented uncertainty characteristic of the pandemic.

**Conclusion:**

Early economic growth projections show that there will be no ordinary recovery for the world economy since short-term countries’ recovery paths are different. It is likely to remain uneven and depend on the effectiveness of the vaccination process, fiscal policy support, public health management, and hard-hit sectors’ growth size in economies. Due to the uncertainty and lack of confidence, governments should ensure an equal and sustainable economic recovery from the Covid-19 pandemic by conducting flexible monetary and fiscal policies. However, without structural reforms, economies can not boost either in the short-term and long-term.

## 1. Introduction

The Covid-19 pandemic, a new strain discovered in China in December 2019, has killed millions of people and transformed the world forever. It is a historic event since it is not just a health issue; it also has global economic, political, and social dimensions. As of August 2021, the virus had infected more than 205 million people worldwide, resulting in around 4.3 million deaths, and more than 2.4 billion vaccine doses have been administered globally [1]. Besides, the International Monetary Fund (IMF) indicates that the global economy was experiencing its worst crisis with a 3.5% global gross domestic product (GDP) fall in 2020 since the Great Depression of the 1930s [2, 3] compared to an estimated 15% GDP decline in between 1929 and 1932 worldwide [4]. While output in the United States (US) declined by 3.5%, the economy contracted by 6.7% and 4.7% in the euro area and Japan, respectively, in 2020. The pandemic is estimated to have pushed 119–124 million people into poverty in 2020 due to the global economic recession [5].

Many scientific comparisons have been drawn between the Covid-19 pandemic and preceding pandemics (Spanish flu, Asian flu, Hong Kong flu, and swine flu) [6] to demonstrate the magnitude of mortality rate and economic collapse caused by the Covid-19 pandemic. However, considering their worldwide dissemination, nature, intensity, and socioeconomic characteristics, drawing comparisons between them can be difficult. The mortality impact of the Covid-19 pandemic, for example, will be less than the Spanish flu that is estimated to have killed roughly 40 million people globally in 1918 [7, 8]. Furthermore, despite the lack of economic data for analyzing the economic impact of the Spanish flu, it is estimated that GDP and consumption fell by 6% and 8% in the typical country, respectively, and that many businesses, particularly those in the service and entertainment industries, suffered significant amount losses in revenue [8, 9]. On the other hand, existing studies show that the global death number of the Asian flu in 1957 and the Hong Kong flu in 1968 was around one million people. Moreover, both pandemics had no significant worldwide economic impact [10, 11]. As a result, early indications suggest that the Covid-19 pandemic will have a similar global impact with the Spanish flu rather than others in the end.

Covid-19 pandemic is a global crisis, and all countries have been affected by this crisis. Developed as well as emerging and developing countries are experiencing a recession. Countries whose economies substantially rely on tourism and hospitality, travel, and entertainment sector have been particularly hard hit. For example, the global GDP loss from the pandemic crisis could be around 9 trillion dollars over 2020 and 2021, greater than the economies of Japan and Germany combined [2]. Moreover, with inferior health systems, smaller fiscal support, and high debt levels, both emerging and developing countries and low-income countries have faced extra challenges for recovery from that noticeable recession.

At the beginning of the Covid-19 pandemic, global uncertainty was at an all-time high, and it continues to be so. Although global economic and policy uncertainty has decreased by about 60% since the onset of the Covid-19 pandemic in the first quarter of 2020, the World Uncertainty Index (WUI) shows that it is still about 50% higher than its historical average from 1996 to 2010 [12]. Hence, the Covid-19 pandemic’s uncertainty is unprecedented since there is a great deal of uncertainty about almost every aspect of the Covid-19 crisis [11]. These are labeled as the virus’ infectiousness and lethality [13], the time required to develop and deploy effective vaccines [14], the duration of social isolation [15, 16], macroeconomic consequences, and government policy responses in both the short-term and long-term [17], the shifts in consumer spending patterns, travel, logistics, new business and working formation [18, 19]. This situation suggests that the global economy will not recover regularly; in other words, the time it takes for each country to recover will most likely vary due to the effectiveness of the vaccination process and public health care measurements implemented by each country. For example, according to the Organisation for Economic Co-operation and Development (OECD), much of Europe will take nearly three years, whereas Korea and the US are likely to recover to prepandemic per capita income levels in roughly 18 months [3]. As a result, the most confusing impact of the Covid-19 pandemic on society, economics, and policies is unprecedented uncertainty since how the pandemic will evolve and end is still ambiguous. 

The Covid-19 crisis demonstrates that governments, not markets, are the ones that provide much-needed help during the global economic recession. In other words, the Covid-19 pandemic has led to a collapse of the free market phenomena, indicating markets are the only solution mechanism for practically every problem that societies encounter since 1981. Nevertheless, the Covid-19 pandemic has promoted the governments’ intervention rather than the market intervention. Almost every major industry has sought financial aid from the government during the pandemic. Moreover, small enterprises have been pleading for zero-interest loans, tax cuts, and outright cash. As a result, the Covid-19 pandemic has demonstrated that markets alone cannot recover economies in this global crisis, and more market and government collaboration will most likely be new economic policy in the postpandemic world [20].

Already existing disparities and gaps in health and social protection systems between countries have been severely revealed, and in many cases worsened, by the Covid-19 pandemic. Countries with strong health and social protection systems responded better to the crisis by guaranteeing access to health care services, also providing jobs and income security for the neediest, such as informal workers, daily wage earners, self-employed workers, migrants, and the homeless. Countries that do not have robust health and social protection systems, on the other hand, have required international assistance to enable an adequate initial reaction to the pandemic. In this respect, the Covid-19 pandemic presents a chance for countries to prioritize investments in their health and social protection systems and develop them to better deal with future crises [21].

Population mental health has deteriorated significantly since the start of the Covid‑19 pandemic. The OECD shows that rates of anxiety and depression increased in 2020 compared to previous years [22]. Economic insecurity, unemployment, lower-income, death fear, domestic violence, mobility restrictions, media exposure about the pandemic, and social isolation have been the main factors that has led to an unprecedented worsening of population mental health during the Covid-19 pandemic [22, 23]. Due to these risk factors, loneliness and individualism are likely to have associated with the pandemic [24]. As individuals are isolated from social life, they have begun to behave more individually than before. People have started to form their own living spaces for conducting their socioeconomic lives based on personal freedoms with the accelerated digitalization in all areas of life. Hence, populations’ social well-being, and their social life and relationships have worsened noticeably during the pandemic. Despite the new positive developments like vaccines, many are still wondering how the postpandemic world will be like.

Governments face formidable difficulties in their efforts to safeguard their citizens from the threat of the Covid-19 pandemic. It is recognized that society’s regular functioning cannot be maintained, particularly in light of the virus’s primary protective measurement, namely confinement. Furthermore, it is acknowledged that the imposed measurements will invariably intrude on rights and freedoms that are an essential feature of a democratic society ruled by law [25]. Countries have no choice but to take extraordinary steps to overcome the pandemic’s unusual situation and save lives, like extensive lockdowns enacted to slow virus transmission and restrict freedom of movement. Such policies may have an unintended impact on people’s lives and security, and access to health care, food, water, sanitation, work, education, and leisure [26]. Furthermore, more security measures may damage democratic principles and fundamental human rights in communities and countries [6]. As a result, the Covid-19 pandemic might lead to a rise in authoritarianism at the global level. 

Our daily lives have forever changed by the Covid-19 pandemic and the resulting economic crisis. One of the most notable developments has been the acceleration of the movement to digital payments, as customers avoided using cash for fear of spreading the virus, and retailers responded by moving their operations online. For example, in 2019, the overall number of noncash payments in the euro area climbed by 81% to €98 billion from €90.7 billion in 2018, and card payments accounted for 48% of total noncash payments in the euro area in 2019 [27]. In total, the global digital payments industry hit more than $4.7 trillion value in 2019 and increased to $5.4 trillion value in 2020, almost a 16% rise compared with the previous year. The entire sector is expected to continue its impressive growth in 2021, with over $6.6 trillion transaction value. According to the growth rates in 2020, while Europe was the leading digital payments market with the 28.3% growth rate to $1.17 trillion compared to 2019, the US market follows with $1.26 trillion worth of digital payments, 22.6% more than the previous year CPA Practice Advisor (2021). Digital Payments to Hit $6.6 Trillion in 2021, a 40% Jump in Two Years [online]. Website https://www.cpapracticeadvisor.com/accounting-audit/news/21208440/digital-payments-to-hit-66-trillion-in-2021-a-40-jump-in-two-years [accessed 19 June 2021]..

Moreover, a cryptocurrency based on blockchain technology, such as Bitcoin and Ethereum, has gained popularity and traction worldwide as a faster and cheaper way to transmit money across borders during the pandemic. Notably, demand for Bitcoin has been surging globally since the beginning of the Covid-19 pandemic. Despite a significant drop in its value in recent months, the value of Bitcoin increased by more than 300% in 2020 Bitcoin.com (2021). Bitcoin Price [online]. Website https://markets.bitcoin.com/crypto/BTC [accessed 10 June 2021].. Today, especially emerging economies, are increasingly turning to cryptocurrencies to help them recover from the pandemic’s adverse economic impacts Oxford Business Group (2021). Economic News [online]. Website https://oxfordbusinessgroup.com/news/can-cryptocurrencies-drive-covid-19-recovery-emerging-markets [accessed 10 June 2021].. On the other hand, central bank digital currency (CBDC) and stablecoins have received more attention recently. According to the Bank for International Settlements (BIS) survey, more than 85% of central banks are studying or investigating CBDC; however, many of issuances have yet to be completed [28]. For example, China, the European Central Bank (ECB) and the Federal Reserve are working to build CBDC CNBC (2021). The Fed this summer will take another step in developing a digital currency [online]. Website https://www.cnbc.com/2021/05/20/the-fed-this-summer-will-take-another-step-ahead-in-developing-a-digital-currency.html [accessed 11 June 2021]. [29]. In contrast to the CBDC, stablecoins are private entities designed to maintain a steady value concerning another asset like a unit of currency and commodity or a basket of assets, unlike cryptocurrencies [30]. Even though the pandemic has highlighted the importance of digital financial services, digital currencies have raised concerns about consumer protection, data privacy, potential cybersecurity risks, disrupting bank lending, and erasing local liquidity from bank deposits [31].

Countries have used big data to combat the Covid-19 pandemic, which enhanced the effectiveness of their efforts in pandemic monitoring, virus tracking, prevention, control, and treatment, as well as resource allocation [32]. However, while using big data to fight the Covid-19 pandemic may improve health care services and their performance, it might also raise other issues related to personal data protection. Before the pandemic, the use of personal data by governments without the permission of individuals was a point of debate; however, it has now become a focal point for human rights violations with the pandemic’s severe measurements.

In line with the situations and discussions mentioned above, it is crucial to study the impact of the Covid-19 pandemic on the world and Turkish economies, mainly because of its massive destruction in all areas. While many studies have examined the global economic consequences of the Covid-19 pandemic [33, 34, and 35], some of them investigated the only Turkish economy [36, 37, and 38]. Furthermore, this research might be viewed as an updated version of our previous work, which examined only the early stages of the pandemic [6]. Both studies might be considered complementary, this one investigates the impact of the pandemic on the global and the Turkish economies from the beginning to the present in contrast to the previous one. Hence, the main objective of this study is to evaluate the potential short-term macroeconomic impacts of the Covid-19 pandemic on the world and Turkish economy based on reliable data released by the IMF, the OECD, the World Bank (WB), Turkish public institutions, and other international institutions and current debate. Analyzing the Covid-19 pandemic impact on the economies in the medium and long term is not our priority since it is not known where the pandemic will evolve.

Nevertheless, every data related to the world economy could not be reached due to the dynamic structure of the pandemic since the national data generally has not been published in the middle of the year. Hence, the international economic organizations could not access some countries’ data. In this context, our study focuses on average OECD area data if the world value of the related economic variable does not exist. Since the OECD economies share in world GDP is around 50% with 38 member countries Organisation for Economic Co-operation and Development (2021). OECD share in world GDP stable at around 50% in PPP terms in 2017 [online]. Website https://www.oecd.org/sdd/prices-ppp/oecd-share-in-world-gdp-stable-at-around-50-per-cent-in-ppp-terms-in-2017.htm [accessed 21 June 2021]., it is thought that average OECD data can be used as a proxy for the world economic data in this study.

As a result, some basic macroeconomic variables such as growth rate, inflation, interest and unemployment rate, trade volume, fiscal balance, travel and tourism, health spending, and fiscal support are presented to analyze the global and Turkish real sector, financial sector, public sector, labor market, foreign trade, and travel and tourism sector developments in the short-term. Furthermore, based on current data and discussions, some assumptions are made concerning possible changes in the global and Turkish economies. Finally, this study is concluded.

## 2. Global economic costs of the pandemic until mid-2021

The OECD reveals that global output fell by 3.5% in 2020 due to a sharp decline in global economic activity [3]. Despite new virus outbreaks in several economies in the fourth quarter of 2020, the global economy recovered faster than projected, and global output remained roughly better than an estimated 4.2% contraction [39]. It should be emphasized that sector specialization in different economies has led to variations in countries’ output growth rates. Those most have relied on international travel and tourism sector faced a more considerable GDP loss in 2020. Global GDP growth is forecast to be 5.8% in 2021 and 4.4% in 2022, with global output exceeding the prepandemic level (% 2.7) by mid-2021. However, global income will still be around $3 trillion, roughly equal to the size of the entire French economy, lower by the end of 2022 than forecast before the crisis [3].

Before the Covid-19 pandemic, the travel and tourism sector employed 10.6% of the worldwide workforce (334 million), but 62 million jobs were lost, representing a drop of 18.5% in 2020. In addition, the global GDP contribution of this sector fell from 10.4% ($9.2 trillion) in 2019 to 5.5% ($4.7 trillion) in 2020 due to ongoing mobility restrictions. Hence, the loss of travel and tourism sector was almost $4.5 trillion in 2020 [40]. Furthermore, destinations worldwide welcomed one billion fewer international arrivals in 2020 than in the previous year. International arrivals dropped by 74% due to an unprecedented fall in service demand and widespread travel restrictions. Also, the collapse in international travel represents an estimated loss of $1.3 trillion in export revenues, which is more than 11 times the loss recorded during the 2009 global economic crisis [41]. As of 2019, international visitor expenditure totaled $1.7 trillion, accounting for 6.8% of total exports. While domestic visitor expenditure fell by 45%, international visitor expenditure fell by a staggering 69.4% in 2020 [40]. Government retention plans and reduced hours support many jobs, but the potential of job losses and contraction persists without full recovery of the travel and tourism sector. 

The International Civil Aviation Organization (ICAO) reveals that global passenger traffic declined by 60%, and the revenue loss was nearly $371 billion in 2020 because of the widespread lockdowns, border closures, and travel restrictions worldwide. Moreover, the estimated decline in total world passengers will be between 44% and 49%, and approximately $289 to 323 billion loss of revenues of airlines is projected for the year 2021 compared to 2019 levels [42]. Nevertheless, cargo flights increased 40% in April 2020 in contrast to the fall in passenger traffic. Also, air cargo demand continued to outperform pre-Covid-19 pandemic levels, with demand up 9% in February 2021 compared to February 2019 level [42, 43]. 

The International Labour Organization (ILO) announces that 8.8% of global working hours were lost relative to the fourth quarter of 2019, equivalent to 255 million full-time jobs in 2020, approximately four times greater than during the global financial crisis in 2009. In total, there were unprecedented global employment losses in 2020 of 114 million jobs relative to 2019. In contrast to previous crises, employment losses in 2020 translated mainly into rising inactivity rather than unemployment, leading to an additional 81 million people shifting to inactivity alongside 33 million additional unemployed. Hence, the unemployment rate rose by 1.1% points to 6.5% around the world in 2020 [44]. Similarly, the unemployment rate increased from 5.4% in 2019 to 7.1% in 2020 in the OECD countries [3]. Before taking into account income support measurements, global labour income in 2020 is estimated to have declined by 8.3%, which amounts to $3.7 trillion, or 4.4% of global GDP [44].

Overall, world trade volumes are expected to increase to 8.2% in 2021, after falling by 8.5% in 2020 despite ongoing weak services trade due to travel restrictions and lack of travellers’ confidence [3]. Similarly, global merchandise trade volume will likely rise by 8% in 2021 after declining by 5.3% in 2020 [45]. In the third quarter of 2020, shipments of computers and electronic components increased by 11%, while textile shipments increased by 24%, boosted by demand for face masks and other protective equipment compared to the second quarter of 2020. Surprisingly, after growing at a 10% annual rate in the first half of 2020, global pharmaceutical exports fell by 1% in the third quarter of 2020, mainly due to summer stockpiling [5]. Hence, the increased demand for digital technology and medical supplies has boosted global trade above prepandemic levels [3]. The better-than-expected performance at the end of the year can be attributed in part to the November release of additional Covid-19 vaccines, which helped to increase business and consumer confidence. On the other hand, commercial services exports fell by 20% in 2020 due to international travel limitations that impeded the delivery of services that required physical presence or face-to-face interaction [45].

Since oil is a crucial intermediate good, particularly for manufactured products and the energy sector, fluctuations in the oil market have a spillover effect. Because of the general recession of the world economy and the decline in demand for fuels and gasoline due to travel restrictions, the Covid-19 pandemic has had a significant impact on global oil demand. Oil prices fell from $67.3 per barrel in December 2019 to $18.4 per barrel in April 2020, but with a steady downward trend in Covid-19 cases in the second half of 2020, oil prices improved as well, pushing the price of Brent oil to around $50 by December 2020 [5].

 Inflation rate in OECD countries was 1.5% in 2020 and is expected to reach 2.7% for 2021. The inflation rate is projected to increase significantly due to the past rise in commodity prices, particularly oil, and some one-off effects of the crisis. A combination of possible negative supply-side effects such as higher operating costs due to containment measurements, disruptions in global supply chains, and a desire to make up for past losses in revenues could push up inflation by more than projected. Upside risks to inflation include further exchange rate depreciation, and food and energy price increases, especially in countries where central bank credibility has already been weakened [3].

Global stock markets have fallen sharply as investors continue to worry about the broader uncertain economic impact of the pandemic. The FTSE, Dow Jones Industrial Average, and the Nikkei all saw massive declines in the first months of the Covid-19 pandemic. Although the major Asian and US stock markets recovered following the announcement of the first vaccine in November, the FTSE is still in negative territory. The FTSE dropped by 14.3% in 2020, its worst performance since 2008 BBC (2021). FTSE 100 suffers worst year since financial crisis [online]. Website https://www.bbc.com/news/business-55500103 [accessed 11 June 2021].. 

The rise in fiscal deficits has stemmed primarily from the collapse in revenues caused by lower economic activity. In response, central banks in many countries reduced the interest rates to make borrowing cheaper and encourage spending to boost the economy. The longer the pandemic lasts, the greater the challenge is to public finances, and government deficits and debt have risen to unprecedented levels. To calm down markets and encourage spending, central banks have lowered policy rates and purchased government bonds, thereby, facilitating the fiscal responses to the pandemic. The size, composition, and duration of fiscal support have varied across countries, which has influenced its effectiveness. Among economies, the majority of supports was devoted to employment protection and household income support [46]. As a result, the fiscal balance increased from a deficit of 3.1% of GDP in 2019 to 10.8% in 2020 in OECD countries. The estimated deficit value for 2021 is 10.1% due to the continuing fiscal supports [3]. Global additional spending and foregone revenues of governments was 9.2% of 2020 GDP [46].

Along with governments, some international financial institutions provide financial supports to economies to rapid recovery from the Covid- 19 pandemic. For instance, the ECB has introduced a Pandemic Emergency Purchase Programme (PEPP) to support the euro area banks, firms, and households through the Covid-19 crisis. In this context, PEPP increased from €500 billion to €1850 billion in December 2020 [5]. In addition, the IMF is providing financial assistance and debt service relief to member countries, facing the economic impact of the Covid-19 pandemic. Overall, the IMF is currently making about $250 billion, a quarter of its $1 trillion lending capacity, available to member countries [47]. Furthermore, the WB has expected to deploy up to $160 billion over 15 months through June 2021 to support countries’ responses to the Covid-19 crisis [48].

Around the world, an estimated 400 million people do not have access to basic health care services. Nearly 100 million individuals are pushed into extreme poverty each year due to having to pay for their own health care. These numbers have increased with the Covid-19 pandemic, as they will continue to rise as people lose jobs and health insurance costs increase [49]. Before the onset of the Covid-19 pandemic, average health spending as a share of GDP across the OECD countries was around 8.8% [50]. Governments have increased their health spending with additional spending or foregone revenues during the Covid-19 pandemic, which is accounted for 1.2% of 2020 GDP. The IMF projected that while average advanced economies’ share of health spending in GDP will increase by 2.6% in between 2020 and 2030, this share will be estimated to be 0.5% and 0.1% for emerging market and middle-income countries, and low-income countries, respectively [46]. Hence, it seems that current inequalities in health care services worldwide will continue in the postpandemic period.

Briefly, as shown in Figure, global economic output is projected to rise by nearly 6% in this year, an impressive surge after the 3.5% contraction in 2020. While the unemployment rate in OECD countries is estimated to fall from 7.1% in 2020 to 6.5% in 2021, there will be no significant change in the fiscal balance deficit of the OECD area, and it is expected to be around 10% of GDP level 2021. Nevertheless, world trade volumes are projected to increase by close to 8.2% in 2021, falling by 8.5% in 2020. Although inflation in the OECD area declined by 0.4% point to 1.5% in 2020, it is expected to rise 2.7% in 2021 due to the delayed higher commodity prices [3].

**Figure:**
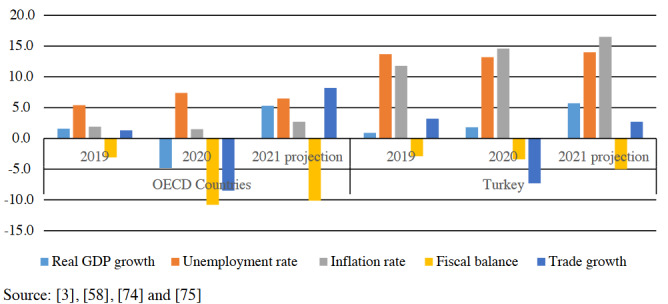
Economic outlook on OECD Countries and Turkey.

## 3. Costs of the pandemic on Turkish economy until mid-2021

Despite the OECD’s prediction of a 1.3% decrease in Turkish GDP in December 2020, Turkey demonstrated a more remarkable recovery from the Pandemic in 2020, with a growth rate of 1.8%. Turkey’s GDP is expected to rise at a rate of 5.7% in 2021 before slowing to 3.4% in 2022 in the absence of possible future shocks [3]. Turkey’s GDP increased by 7% in the first quarter of 2021 [51], but the surge in infections which appears to have peaked in May 2021. Therefore, new confinement measures have affected employment, incomes, and private consumption from the second quarter of 2021. The vaccine rollout started fast in February, but the authorities ran into serious procurement issues, causing them to scale down their goals and seek more diverse measurements until June 2021 [3].

Turkey’s overall export and import values were nearly $169.482 billion and $219.397 billion, respectively, resulting in a negative trade balance of $49.915 billion in 2020, which is higher than the $29.512 billion negative trade balance in 2019 [52]. Turkey’s net exports declined by 7.3% in 2020 but it is expected to increase 2.7% in 2021 [3]. In May 2021, exports increased by 65.50%, and imports increased by 54% compared to the previous year’s same month [53]. As of 2020, the top export destinations of Turkey were Germany (9.4%), the US (6%), and Italy (4.7%) and the top import origins were China (10.4%), Germany (9.8%), and Russia (8.1%) [52]. These data show that the supply chain of Turkey does not depend on one country primarily, so the Covid-19 pandemic’s detrimental influence on the manufacturing sector did not persist long in Turkey. For instance, the manufacturing sector in Turkey shrank by 8% in the second quarter of 2020, but then this sector showed a strong rebound in the next quarter with a 28.6% growth [51]. Therefore, the pandemic has not hurt the Turkish manufacturing sector as expected in the end. Turkish current account deficit recorded $1.127 million, indicating a decrease of $1.947 million compared to June of the 2020, bringing the 12-month rolling deficit to $29.674 million [54]. Although Turkey had a current account surplus in 2019 with 0.9% of GDP value, the Covid-19 pandemic widened the current account deficit to 5.2% in 2020 [3]. However, this deterioration was mainly driven by a decrease in the goods trade deficit and an increase in services surplus, which results from the global economic disruptions caused by the Pandemic.

The tourism sector has been hardly damaged in Turkey since tourism income fell by 65.1% and declined to $12 billion in 2020 compared to the previous year. In parallel, departing visitors decreased by 69.5% in 2020 compared to 2019 and declined to nearly 16 million people [55]. In April 2020, global international passenger capacity experienced an unprecedented 94% reduction for Turkey [42]. The navigational charge losses of Turkey were approximately $210 million in 2020, declined from $363 million in 2019 to $153 million at the end of 2020 [56]. Additional measures to facilitate a strong tourism season in 2021 summer have been implemented in Turkey, including a vaccination process for employees of the travel and tourism sector.

Turkey has struggled with the high unemployment rate (13.5%) in the first quarter of 2021 since 2018. The number of unemployed persons increased by 89 thousand to 4 million 277 thousand persons compared to the same quarter of the previous year [57]. It seems that the unemployment rate increased, especially among the blue-collar workers and service sector employees, due to the bankruptcies and closures of factories and small and medium workplaces, who have been probably suffered the major economic losses of the Covid-19 pandemic. This situation has led to income losses for workers because of the weak labour market in Turkey. 

Moreover, the Consumer Price Index (CPI) of Turkey increased by 16.59% annually in May 2021. On the other hand, transportation with 28.39%, furnishings and household equipment with 21.79%, and health with 19.30% were the main groups where high annual increases were realized [58]. However, the central bank’s management has reiterated its commitment to the 5% inflation target against the 16,59% current inflation rate and persisting inflationary pressures in Turkey [59].

Additionally, unprecedented uncertainty will bring more risks for investors in Turkey. The value of Turkey’s Economic Confidence Index, 99.3 in January 2020, decreased by 3.1 points to 96.2 in January 2021. This fall stemmed from the decline in service and retail trade confidence indices [60]. On the other hand, the Turkish Real Sector Confidence Index increased from 62.30 to 107.40 in April 2021 compared to the same month in the previous year [61]. Therefore, the data indicate that increased confidence will bring better business performance in Turkey soon.

The Central Bank of the Republic of Turkey (CBRT) decided to keep the policy interest rate at 19% in May 2021 to improve the financial conditions, which is higher than compared to the same month in the previous year (8.25%) [62]. Furthermore, on March 31, the CBRT introduced a program of outright purchases of sovereign bonds and has substantially increased its liquidity facilities to banks [63]. Today, Turkey’s 5 Years Credit Default Swaps (CDS) premium value is high with 382.62 points on 21 June 2021 but lower than 2020’s maximum value of 643.15. This can be interpreted as the Turkish economy is under the pressure of financial risk World Government Bonds (2021). Turkey 5 Years CDS [online]. Website http://www.worldgovernmentbonds.com/cds-historical-data/ [accessed 13 June 2021]. since the higher CDS premium might cause pressure on the Turkish foreign borrowing interest rate to rise. 

The Turkish authorities predict that the total discretionary fiscal support package will cost 638 billion Turkish liras, 12.7% of GDP, in March 2021 to fight the Covid-19 pandemic. Debt guarantees to businesses and people, loan service deferrals by public banks, tax deferrals for businesses, equity injections into public banks, and a short-term labor program are examples of crucial fiscal measurements in Turkey [63]. In addition, Turkey implemented substantial Value-Added Tax (VAT) cuts for services and a withholding tax reduction for tradespeople. For instance, the VAT on passenger transportation, wedding organizations, residential maintenance and repair, dry cleaning, and tradespeople services like tailoring was reduced from 18% to 8%. Moreover, until mid-May 2021, a nationwide prohibition on layoffs was in effect. Besides, for families with monthly salaries of less than 5.000 Turkish liras ($740), state lenders have proposed a low-interest credit package of up to 10.000 Turkish liras ($1.477). The government also announced that it would pay 60% of the staff salaries of firms forced out of business under the short-term employment allowance program. In addition, the minimum pension was raised to 1.500 Turkish liras ($221) to protect retirees from the pandemic’s harmful consequences, and bonus payments were pushed to earlier dates. The government has recently begun paying 1.000 Turkish liras ($148) to 4.4 million needy families [64]. In the first quarter of 2021, compared to the same quarter the previous year, employee compensation climbed by 16%, while net operational surplus/mixed-income increased by 39.1% [57].

Among G20 emerging economies, Turkey provided the most liquidity support compared to its GDP in response to the Pandemic. Turkey left behind countries, including China, Brazil, India, and South Africa, with a liquidity support to GDP ratio of 9.5%. Brazil followed it with 6.2% and India with 5.2%. This ratio was 1.5% in Russia, and 1.3% in China. Besides, Turkey’s additional spending and foregone revenues were 2.7% of 2020 GDP and lag behind many countries [65].

Public deposit banks began new retail loan campaigns for home buying and consumer spending in June 2021. Also, loans to farmers have been postponed for six months. As part of the government’s Coronavirus Economic Stability Shield program, the Treasury-Backed Credit Guarantee Fund increased from 25 billion Turkish liras ($3.67 million) to 50 billion liras ($7.34 million). The enterprises’ principal and interest payments were postponed for at least three months, and public lenders refinanced them. Turkey extended repayment periods for certain credit card loans, introduced low-interest credit packages for low-income households, allowed tradespeople to postpone payments without penalty, provided new low-interest loans and credit cards with longer repayment periods for tradespeople, and offered new credit packages for their jobs [66].

Turkey’s health spending was roughly 4.4% of its GDP in 2019, lower than the OECD average of 8.8%. However, health spending in OECD countries increased sharply in 2020 due to the pandemic. According to preliminary estimates, health spending in a group of 16 OECD countries jumped to roughly 9.9% of GDP on average in 2020 [50]. Turkey has also boosted its health spending through additional spending or revenue foregone during pandemic, accounting for 0.3% of GDP in 2020. As a result, IMF estimated that Turkey’s health spending as a share of GDP would rise 0.5% on average until 2030 [46]. 

Overall, Turkey is the 19^th^ largest economy globally, with a GDP of $761 billion [67]. Turkey was among the few countries to experience positive economic growth in 2020. As shown in Figure, GDP growth is expected to be strong in 2021 with 5.7% if there will be no unprecedented shocks. Turkey’s unemployment rate decreased by 0.6% point to 13.1% in 2020 due to the decline in labor force participation. It is estimated to increase 14% in 2021 due to the weak labour market during the Covid-19 pandemic [3]. Besides, inflation rate increased by 2.8% point to 14.6% in 2020, probably due to inflation expectations and risk premia. Inflation is projected to rise to 16.5% in 2021 in Turkey [68]. Turkey’s net export volume fell from 3.2% in 2019 to -7.3% in 2020, and it will be expected to rise to 2.7% in 2021 as in other countries [3]. On the other hand, Turkey’s fiscal balance deficit rose from 2.9% of GDP in 2019 to 3.4% in 2020. However, the Turkish fiscal balance will be worsened by an estimated 5% deficit in 2021 [68]. 

## 4. Discussion

Estimating the real economic consequences of the Covid-19 pandemic is currently difficult due to the Pandemic’s spiral effects on both the national and global economies as a result of increased trade and financial linkages brought on by globalization [69]. In general, the crisis response is organized around four thematic pillars that are aligned with economies’ comparative advantages: saving lives threatened by the pandemic, protecting the poor and vulnerable, assisting in the retention of jobs and businesses, and working to build a more strong recovery [48]. Countries who were fast to vaccinate their populations against the Covid-19 pandemic and manage infections through efficient public health measurements will likely rebound more quickly. However, while vaccination rates in many advanced economies increase, poorer and emerging market countries lag behind [3]. In the short term, global economic recovery will be impossible if equal vaccination distribution between and within countries is not achieved. For example, only 1.2% of people in low-income countries have gotten at least one dose of the Covid-19 vaccination, even though 31.2% of the world’s population has received at least one dose Our World in Data (2021). Statistics and Research: Coronavirus (COVID-19) Vaccinations [online]. Website https://ourworldindata.org/covid-vaccinations [accessed 16 August 2021].. This divergence will probably tend to increase economic inequality within countries in the short-term. However, this condition might hurt the world’s social peace in the medium and long-term.

It is crucial to highlight that in the early stages of the Covid-19 pandemic, the global leader of the US failed to safeguard its citizens, leaving them unwell and ruined. Millions of Americans have become impoverished and unable to access health care services. The Covid-19 pandemic killed nearly as many Americans than all of the military conflicts of the last 70 years combined Financial Times (2021). Covid-19 looks like a hinge in history [online]. Website https://www.ft.com/content/de643ae8-9527-11ea-899a-f62a20d54625 [accessed 14 June 2021].. Despite warnings and considerable advantages, including vast resources, biomedical infrastructures, and scientific skills, the US missed every opportunity to contain the virus. In contrast to many other countries, refused to take effective measurements to reverse the virus’s upward trend. This delay has affected the American economy badly, and the US economy contracted by 3.5% in 2020 [3]. According to the US experience, acting quickly is likely the most critical lesson in determining national outcomes during pandemics.

Although increasing vaccine production and distribution is the most substantial current economic policy for boosting economic development, the future of the global economy remains uncertain, and the recovery will be uneven. According to the IMF’s economic predictions, between 90 million and 130 million full-time equivalent jobs will be lost, with global output growth of about 6% in 2021. However, this estimation is subject to vary based on virus evaluations around the world. Thus, the picture is highly unpredictable, with both upside and downside risks making forecasting difficult. Therefore, rebuilding confidence in macroeconomic policy and structural reforms are vital for rebounding the national and world economy [70].

The Covid-19 pandemic demonstrates the importance of public health management. However, many nations’ health systems are overburdened, and expenditures will be required to improve staff and health care capacity to manage the possibility for Covid-19 renewal and subsequent pandemics. Governments all over the world, on the other hand, have promised billions of dollars towards a Covid-19 vaccine and treatment options; as a result, the market shares of some pharmaceutical companies involved in vaccine development have increased [71]. As a result, it is not incorrect to suggest that pharmaceutical corporations have benefited from the Covid-19 pandemic at most. In addition, several countries supply vials, syringes, needles, and even cool boxes and freezers needed to manufacture, distribute, and administrate vaccines. Hence, mass production, distribution and administering the vaccine supply chain are essential as vaccination [3].

ILO reveals the contrast between massive job losses in hard-hit sectors such as accommodation and food services, arts and culture, retail, and construction and the positive job growth evident in many high-skilled services sectors such as information and communications technology (ICT), and financial and insurance activities [44]. The World Economic Forum’s Future of Jobs Report 2020 projected that technological change is set to displace a range of skills in the labour market while driving greater demand for a new set of core skills such as analytical thinking, creativity, critical thinking, and digital skills [72]. Because of a particular shortfall in digital skills, there is a significant additional disruption in the labour market due to the Covid-19 crisis. Also, only 53.6% of the global population uses the internet, which refers to the digital divide around the world. Hence, the impact of the pandemic should serve as a wake-up call for countries that need to embrace the digitalization process, incentivize companies to move towards digital business models, and invest more in ICT development and digital skills [73]. 

The Covid-19 pandemic measurements also disrupted global education worldwide, and over 1.6 billion students in more than 190 countries were out of school in months. Two-thirds of an academic year has been missed on average worldwide due to full or partial closures in months, while half of the world’s student population is still affected by closures. The long-term closure of schools poses a great risk for students’ future since they will probably fall below the minimum proficiency levels in both theory and practice. Besides, 24 million children and youth are at risk of dropping out due to the digital divide. Since digital transformation is closely associated with human capital development, more education funding is needed for education recovery in most countries to improve online education services and upgrade curricula for changing world [74]. Also, more alignment is needed between employers and educators to enable students to get the new digital economy skills. However, these reforms will probably bring additional economic costs to the fiscal budget of countries.  

For many economies, currently, debt affordability is not a risk, but it will be inevitable due to the high amount of income support and government support packages. Vaccine supply brings an extra economic burden to the budget of countries. These additional spendings will bring more taxation on consumers and producers in the future. This situation is likely to deepen the economic crisis in the medium and long-term. Today, international financial institutions like IMF and WB provide financial assistance and debt service relief to member countries facing the economic impact of the Covid-19 pandemic. Nevertheless, whether these aids are given equal or transparent between countries is a separate debate. 

There will inevitably be paradigm shifts in the economy in the postpandemic period. In contrast to the free market economy, public and private sector cooperations will be essential for economic activities since the Covid-19 pandemic shows that neither governments nor businesses can achieve this complex postpandemic economic transformation alone. In addition, it seems the well-balanced fiscal support and flexible approach need to the economic recovery with the cooperation of the public and private sectors [75].

Today, global viruses will be seen as a threat like a nuclear attack, biological weapon, or global terrorism since their impacts on humans and the world are similarly very destructive, as seen in the Covid-19 pandemic [6]. Furthermore, the pandemic has brought to light another potential global threat, named bioterrorism, because the virus’s terrible impact, which these terrorist groups have acknowledged, has renewed their interest in acquiring, manufacturing, and employing biological weapons [76]. For this reason, countries that could develop a biological defense system or infrastructure for the virus attack will have more power in the postpandemic world. 

## 5. Conclusion

Although there were variations in recovery across economies, Turkey was among the few countries with China that showed a solid and positive rebound with 1.8% economic growth during the Covid-19 pandemic in 2020 though the global output declined by 3.5%. Hence, Turkey is apart from its peers due to the rapid recovery resulting from initial better policy responses such as monetary and credit expansion and large liquidity support. From this perspective, it is expected that the world and Turkey’s GDP growth will be 5.8% and 5.7%, respectively, in 2021 without further major shocks. Nevertheless, new confinement measures taken in May 2021 due to the pandemic’s third wave might adversely affect employment, incomes, private consumption, and the travel and tourism sector in Turkey. This unprecedented uncertainty characteristic of the Covid-19 pandemic has likely changed all economic predictions, and sustainable growth for Turkey might not be possible in the short-term. 

Global economic output is projected to rise by nearly 6% in 2021, an impressive surge after the 3.5% contraction in 2020, but there will be no ordinary recovery in the world due to the unprecedented economic uncertainty. Also, economic rebound depends on the effectiveness of vaccination programs, public health policies, fiscal polices, and the country’s dependence on a hard-hit sector such as travel and tourism in the short-term. Although the unemployment rate and fiscal balance deficit are projected to fall in 2021 in OECD countries, world trade volumes are estimated to rise. Besides, inflation is expected to increase in the OECD area due to the past rise in commodity and oil prices, and some one-off effects of the crisis. 

In Turkey, as in other countries, the economic impact of the Covid-19 pandemic has been severe. Turkey’s unemployment rate decreased due to the decline in labor force participation but it is estimated to increase due to the weak labour market during the Covid-19 pandemic. Besides, poverty in Turkey is estimated to have risen by about 1.5 million people as a result of the pandemic [68]. Moreover, the inflation rate has increased, most probably due to the inflation expectations and risk premia. It is obvious that Turkey’s unemployment and inflation rate displayed a different trend in 2020 compared to the OECD economies. Although the tourism sector has been hardly damaged in Turkey, a sharp increase in tourism revenues is expected in 2021 once mass vaccination occurs. As of August 2021, the share of people fully vaccinated against Covid-19 virus is 39%, and the share of people only partly vaccinated against Covid-19 virus is 13% in Turkey, and Turkey ranked 17th in the world Our World in Data (2021). Statistics and Research: Coronavirus (COVID-19) Vaccinations [online]. Website https://ourworldindata.org/covid-vaccinations [accessed 16 August 2021].. 

Although Turkey’s fiscal balance deficit rose, it showed a better performance than other OECD countries. Turkey provided the most liquidity support among G20 emerging economies during the pandemic. However, the Turkish fiscal balance will be worsened because of the extension of fiscal supports. Currently, the actual costs of the fiscal policy supports and vaccination to the Turkish economy have not been yet precise. 

Today, vaccines are seen as the key to a safe and permanent transition to more typical economic and social conditions. Countries that have been quick to vaccinate their population against the Covid-19 virus and manage to control infections through effective public health strategies have likely displayed more quick recovery. Hence, it seems that the pandemic will provide some economic and political opportunities to some countries if they can manage to control the virus earlier by effective vaccination policy.

As a result of the digital transformation in every industry, the world will not be the same in the postpandemic period. Remote working, video-conferencing, online entertainment, e-commerce, and digital currencies, for example, have all grown in popularity since the beginning of the pandemic. Hence, countries should focus on the digitalization process, dissemination of ICT, and digital skills. Supporting students and young people to remain in education and enabling them to acquire digital skills by upgrading their curriculum based on new labour market demand should be a priority for governments. Countries should take urgent measurements to prevent a youth unemployment crisis and promote better mental health for young people. Moreover, cooperation between public and private sectors will probably be a new economic approach rather than a free market.

In sum, Turkey must concentrate on new structural reforms to boost the economy, invest in health care, adapt to the new postpandemic world, and achieve a sustainable economy along with the other countries. Overall, trends in other economies suggest that the cost of the Covid-19 pandemic will not be recovered just by attaining a vaccination threshold in the short-term. In other words, vaccination seems to be a beginning rather than an end, and the policy choices governments make today will determine their place in the new world. Nevertheless, global economic recovery will be impossible in the near future if equal vaccination distribution between and within countries is not achieved.
